# Nonequilibrium Thermodynamics in Biochemical Systems and Its Application

**DOI:** 10.3390/e23030271

**Published:** 2021-02-25

**Authors:** Dongliang Zhang, Qi Ouyang

**Affiliations:** 1 The State Key Laboratory for Artificial Microstructures and Mesoscopic Physics, School of Physics, Peking University, Beijing 100871, China; dlzhang@pku.edu.cn; 2 Center for Quantitative Biology and Peking-Tsinghua Center for Life Sciences, AAIC, Peking University, Beijing 100871, China

**Keywords:** nonequilibrium thermodynamics, biophysics, stochastic thermodynamics, nonlinear physics, chemical reaction network

## Abstract

Living systems are open systems, where the laws of nonequilibrium thermodynamics play the important role. Therefore, studying living systems from a nonequilibrium thermodynamic aspect is interesting and useful. In this review, we briefly introduce the history and current development of nonequilibrium thermodynamics, especially that in biochemical systems. We first introduce historically how people realized the importance to study biological systems in the thermodynamic point of view. We then introduce the development of stochastic thermodynamics, especially three landmarks: Jarzynski equality, Crooks’ fluctuation theorem and thermodynamic uncertainty relation. We also summarize the current theoretical framework for stochastic thermodynamics in biochemical reaction networks, especially the thermodynamic concepts and instruments at nonequilibrium steady state. Finally, we show two applications and research paradigms for thermodynamic study in biological systems.

## 1. Introduction

How should a living system, e.g., a cell, be described from a physical aspect? In 1943, Erwin Schrödinger provided a systematic point of view in his speech, which explained the basic physical logic of cellular activities. The content, which was quite novel and interesting to people especially those with physical background, was later summarized into a book named *What is life* [1] and became famous worldwide. In this book, he proposed that, the key difference between a living cell and a “dead” system is its ORDER. In a living cell, countless biochemical reactions occur regularly, orderly and endlessly, which would not happen in a non-living system. For a system without living activity, for example, a box of gas mixture, when put into a fixed environment its intrinsic reactions will soon enter the inactive, stable, orderless, dead thermodynamic equilibrium state where all the ordered intrinsic activities will stop and the entropy of the system reaches maximum, whereas living system maintains an ordered and relatively steady state and never reaches equilibrium. Why could this ordered steady state for living systems be maintained? According to the 2nd law of thermodynamics, the entropy of an isolated system would never decrease. Hence, as Schrödinger pointed out, the key reason is that living cells exchange energy and materials with external environment, absorb necessary energy and nutrients for biochemical reactions and expel waste and heat from the reactions. In terms of statistical physics, the system is maintained at a low entropy state to make a living, by absorbing “negative entropy” from environment to nullify the entropy production from its necessary biological activities.

Schrödinger’s point of view had great impact on biology and physics. It has been widely accepted now that metabolism, which describes how a living system continuously consumes necessary nutrients and energy taken from environment for life-sustaining, is the essence of living activities. On the other hand, this point of view first explicitly connected biological phenomenon with the theory of statistical physics, hence drew a large group of statistical physicists’ attention to systems far from equilibrium, i.e., nonequilibrium systems. Despite the success in explaining various non-living phenomena, the traditional equilibrium and near-equilibrium statistical physics had long been difficult to use in living system. After Schrödinger proposed his idea, physicists started to realize that open nonequilibrium systems (represented by living systems) are quite complicated and a new angle was needed.

Another succeeding revolutionary idea comes from the Brussels School. During 1950s, the founder of Brussels School Prigogine and some other researchers discovered the so-called “minimal entropy production principle” [2,3,4] for systems near equilibrium. This principle declares that for systems near equilibrium where the response to thermodynamic force is linear, the steady state (under a certain thermodynamic force) would be the state where entropy production rate takes minimum. However, this principle cannot be used to explain some complicated nonequilibrium phenomena, for example, Rayleigh-Bénard convection [5,6,7,8] and Belousov–Zhabotinsky reaction oscillation [9,10,11,12,13]. These systems are maintained at a highly ordered state, so the entropy production in these systems must be high and should not be at minimum. One common characteristic in these systems is that the response to thermodynamic force is highly nonlinear, and this nonlinearity is why minimal entropy production principle does not apply and ordered patterns arise.

Since Prigogine and colleges found that it’s impossible to apply the minimal entropy production principle to systems arbitrarily far from equilibrium, they proposed the famous dissipative structure theory [14,15]: for systems that are far from equilibrium, the response functions to various thermodynamic forces go beyond linear regime described by linear response theory and arrive at the nonlinear regime. In this regime, the system may be maintained at a relatively steady state (the nonequilibrium steady state) by dissipating free energy and producing entropy, which can exhibit complex and ordered dynamics.

Dissipative structure theory, combined with Schrödinger’s novel idea, pointed out several fundamental requirements for ordered systems including living systems in terms of statistical physics:

1. The system must be open. By exchanging energy with external environment, a system’s ordered steady state is maintained with free energy dissipated and entropy (produced by nonequilibrium activities) expelled. In living systems and other chemical reaction systems, mass exchange is also needed in form of chemical reactants
2. The system must be driven far enough from equilibrium. If the system is at or near equilibrium, it would be described by minimal entropy production principle and not be available for a steady ordered state.
3. The nonlinearity in the system must be strong enough. Week nonlinearity would not lead to a complex and non-trivial dynamics.

When the above requirements are satisfied, self-organized steady ordered structure, i.e., dissipative structure, may arise.

Prigogine’s theory shed light on the research on thermodynamics for system far from equilibrium and its relation with complex nonlinear systems including living systems. With a series of proceeding work since 1980s on nonequilibrium thermodynamics, it’s now possible to explore the thermodynamic rules within living systems.

## 2. The Development of Nonequilibrium Statistical Physics and Stochastic Thermodynamics

Modern statistical mechanics is based on stochastic theory, where the dynamics of a system is described by stochastic process [16,17]. When the system is near equilibrium, its dynamics is simple fluctuation near the equilibrium state. As introduced above, it has been quite clear for long to physicsts how to describe such fluctuation. However, it was hard for physicists to discover the universal rules applicable to systems arbitrarily far from equilibrium, until recent several decades. The landmark results are the so-called Jarzynski equality [18,19], a series of fluctuation theorems [20,21,22,23] among which the most famous one is Crooks’ fluctuation theorem [21], and the so-called thermodynamic uncertainty relation (TUR) [24,25,26,27].

Jarzynski equality describes the relation between the work done and the free energy change during a general isothermal process. When the system is at a unique certain macroscopic state *A*, its Hamiltonian $H_{A}$ is also fixed. When put into a heat bath with temperature *T*, according to equilibrium statistical physics, it corresponds to one certain equilibrium state whose probability of each microscopic state follows Boltzmann distribution, $p_{s}^{A}=Z_{A}^{-1}\exp\left[-\beta H_{A}\left(s\right)\right]$, with $H_{A}\left(s\right)$ the Hamiltonian for the microscopic state *s*, $Z_{A}=\sum_{s}\exp\left[-\beta H_{A}\left(s\right)\right]$ the partition function, and $\beta ={\left(k_{B}T\right)}^{-1}$. The free energy at equilibrium state $F_{A}=k_{B}T\ln Z_{A}$ is also fixed. When the macroscopic state is changed to another macroscopic state *B* by doing work, e.g., pulling a spring, pushing a piston, or changing the conformation of a protein complex, the Hamiltonian would be changed to $H_{B}$. Same as above, macroscopic state *B* also corresponds to a unique certain equilibrium state with free energy $F_{B}=k_{B}T\ln Z_{B}$. According to the 2nd law, in thermodynamic limit, the work done during this process should no smaller than their free energy difference $\Delta F=F_{B}-F_{A}$. However, thermodynamic limit only applies in ensemble sense. For each single realization of this stochastic process, the initial state of the system follows the Boltzmann distribution, and is affected by noise from heat bath. Hence during this process, the system would not always follow the same trajectory in phase space, and the work done in each single realization *W* may also be different. Previous study including the 2nd law actually just stated that the average work done 〈W〉≥ΔF. Jarzynski pointed out that, we should focus not only the average work done 〈W〉, but also the work distribution for each realization, and he found the following equality:

(1) $$\langle e^{-\beta W}\rangle =e^{-\beta \Delta F}.$$

It could also be easily checked with Jensen’s inequality that Equation (1) simply implies the 2nd law 〈W〉≥ΔF.

Jarzynski equality is a great breakthrough to 2nd law, and was confirmed by experiments in 2000s [28,29,30]. For hundreds of years, Jarzynski equality for the first time shows how the 2nd law rules thermodynamic processes in the form of equality and beyond its previous inequality form, and is applicable to systems arbitrarily far from equilibrium.

Crooks’ fluctuation theorem describes the relation between the probability of a trajectory in phase space and the corresponding entropy production [21]. Suppose there is an amount of entropy production ω along one trajectory (forward trajectory), then the corresponding reverse process (reverse trajectory) would naturally produce −ω entropy (absorb ω entropy). Denote the probability of the forward trajectory as $P_{F}\left(\omega \right)$ and the probability of reverse trajectory as $P_{R}\left(-\omega \right)$. When ω>0, according to the 2nd law, in thermodynamic limit $P_{R}\left(-\omega \right)/P_{F}\left(\omega \right)\to 0$, i.e., the process that decreases the overall entropy would never occur. However, in small systems where stochasticity is not negligible, it’s possible to occur with a small probability. This stochasticity is described by Crooks’ fluctuation theorem, which declares that in small systems the ratio $P_{R}\left(-\omega \right)/P_{F}\left(\omega \right)$ is exponentially suppressed by ω, i.e.,

(2) $$\frac{P_{R}\left(-\omega \right)}{P_{F}\left(\omega \right)}=e^{-\omega /k_{B}}.$$

According to this formula, the larger ω is, the more impossible would the reverse process be. This is compatible with the 2nd law, since ω is an extensive quantity and is proportional to the particle number. For a “macroscopic” trajectory, where the particle involved in is around ∼1 mol, the entropy production is in the order of ∼$1\;\mathrm{mol}\times N_{A}k_{B}$∼$10^{23}k_{B}$, so $\omega /k_{B}$ is very large. In this sense, $e^{-\omega /k_{B}}\approx 0$, and the probability of the reverse process is almost impossible. For a small system, ω is finite, so the process that “seemingly violate” the 2nd law would occur more frequently. Such fluctuation resulted from stochasticity is why “fluctuation theorem” gets its name. This quantitative relation between the fluctuation probability and the corresponding entropy production has been confirmed by experiments [31,32,33].

Crooks’ fluctuation theorem, just like Jarzynski equality, is also a breakthrough to the 2nd law. Actually, by considering Jarzynski equality, Crooks’ fluctuation theorem can be expressed in a simpler form:

(3) $$\langle e^{-\omega /k_{B}}\rangle =1.$$

These theories are the most breakthrough in recent 30 years. For the first time, physicists could be able to explore laws in statistical physics far away from equilibrium with these theoretical instruments. They also indicate that, only the statistical laws in mesoscopic systems, where system are small so fluctuation is not negligible but not too small for statistical physics to fail, are fundamental.

Seifert and some proceeding researchers extend these theories together with definition of some other thermodynamic variables to the motion of single particle. They calculated the entropy production rate in this case and proved the corresponding fluctuation theorem [34]:

(4) $$\langle e^{-\Delta s_{tot}/k_{B}}\rangle =1,$$

where $\Delta s_{tot}$ is the entropy production for single particle along a trajectory. This is the same with Crooks’ fluctuation theorem. Meanwhile, there are some other forms of fluctuation theorems, which are catalogued in a review from Seifert [23].

After these work, statistical physics can be applied to a much wider range of studies. Researchers now are able to discuss the systems not only at or near equilibrium, but also the systems arbitrarily far from equilibrium and systems even small to single particle with external stochastic force, in terms of thermodynamics. Thus, a new branch named stochastic thermodynamics [23,34,35,36,37,38,39,40] is established and soon becomes a research hotspot in statistical physics and some related fields.

With the branch established, recently an inequality named “thermodynamic uncertainty relation” (TUR) was proposed [24,25,26,27] which shows the relation between the fluctuation and the entropy production at nonequlibrium steady state, and this relation is one of the most important and impacting theoretical results in the last years. Generally, the microstates of the system could form lots of cycles. At nonequilibrium steady state, there would be cyclic fluxes on these cycles (see Section 3.3 below for more details on cycle theory). Quantitatively, denoting the stochastic net cyclic flux on a cycle as *j* and the corresponding entropy production rate on this cycle as $e_{p}^{s}$, a relation will hold:

(5) $$\frac{\sigma_{j}^{2}}{{\langle j\rangle }^{2}}\cdot e_{p}^{s}\geq 2k_{B},$$

where $\sigma_{j}^{2}$ is the variance of *j* and 〈j〉 is the average of *j*. It’s clear that if the entropy production rate $e_{p}^{s}$ is increased, the lower bound of the relative fluctuation $\sigma_{j}^{2}/{\langle j\rangle }^{2}$ of the cyclic flux could be suppressed. This relation can also be written in the integral form in a finite time interval τ:

(6) $$\frac{\sigma_{j_{\tau }}^{2}}{{\langle j_{\tau }\rangle }^{2}}\cdot \Sigma_{\tau }\geq 2k_{B},$$

where $\langle j_{\tau }\rangle =\langle j\rangle \times \tau$ is on average how many times the cycle is completed in the time interval τ and $\Sigma_{\tau }$ is the entropy produced in τ. These relations are the called thermodynamic uncertainty relations (TURs).

TUR shows how the stochastic dynamic quantities (fluctuations) are related to thermodynamic quantities (entropy production rates). By increasing entropy production rate, the noise of the dynamics may be suppressed, and vice versa. It also reveals that, it’s impossible to reduce the fluctuation and entropy production rate simultaneously to infinitely small. This relation is attracting more and more attention, and is one of the most important theoretical results in this field in the past years.

## 3. Nonequilibrium Steady State in Chemical Reaction Networks and Its Thermodynamics

As introduced above, self-organized dissipative structure will maintain its ordered state for relatively long time, although far from equilibrium. Therefore, thermodynamic rules and properties in such steady states, i.e., nonequilibrium steady states (NESSs), are quite interesting to physicists especially biophysicists. The thermodynamic laws and methodologies in NESS, especially those in biochemical NESS, are of great importance to study the thermodynamic properties of living systems. Here we introduce the current thermodynamic theories in chemical reaction networks.

### 3.1. Description of Chemical Reactions with Stochastic Process

In the framework of stochastic process, chemical reactions are considered as a series of Markov processes described by chemical master equations (CMEs) [41,42]. Consider a general chemical reaction:

(7) $$\sum_{i=1}^{J}s_{i}X_{i}\overset{k_{+}}{\underset{k_{-}}{⇌}}\sum_{i=1}^{J}r_{i}X_{i},$$

where $X_{i},i=1,2,\ldots ,J$ is the *i*-th component and $s_{i},r_{i},i=1,2,\ldots ,J$ the corresponding stoichiometric coefficient. Denoting $n_{i}$ as the molecule number of $X_{i}$, the microscopic state of the system can be represented by $\vec{n}=\left(n_{1},n_{2},\ldots ,n_{J}\right)$. For the forward reaction, after the reaction happens, $n_{i}$ will be changed to $n_{i}+\left(r_{i}-s_{i}\right)$ with the reaction frequency proportional to probability that the reactant molecules collide, which is also proportional to

$$\prod_{i=1}^{J}\frac{n_{i}!}{(n_{i}-s_{i})!}.$$

Similar for the reverse reaction, after the reverse reaction happens $n_{i}$ will be changed to $n_{i}-\left(r_{i}-s_{i}\right)$ the reaction frequency proportional to

$$\prod_{i=1}^{J}\frac{n_{i}!}{(n_{i}-r_{i})!}.$$

Hence, considering other effects such as volume, the corresponding CME describing the evolution of the system’s probability density $P(\vec{n},t)$ can be written as [41]

(8) $$\begin{array}{c} \frac{dP(\vec{n},t)}{dt}=\left\{k_{+}V\left(\prod_{i=1}^{J}E^{s_{i}-r_{i}}-1\right)\prod_{j=1}^{J}\left[\frac{n_{j}!}{\left(n_{j}-s_{j}\right)!V^{s_{j}}}\right]\right.\;\;\;\;\;\;\;\;\;\;\;\\ \left.+k_{-}V\left(\prod_{i=1}^{J}E^{r_{i}-s_{i}}-1\right)\prod_{j=1}^{J}\left[\frac{n_{j}!}{\left(n_{j}-r_{j}\right)!V^{r_{j}}}\right]\right\}P, \end{array}$$

where *V* is the volume and E is the “step operator” defined by its effect on arbitrary function f(n): Ef(n)=f(n+1).

If the system consists of *m* reactions (m>1) forming a chemical reaction network (CRN),

(9) $$\sum_{i=1}^{J}s_{i}^{\rho }X_{i}\overset{k_{+}^{\rho }}{\underset{k_{-}^{\rho }}{⇌}}\sum_{i=1}^{J}r_{i}^{\rho }X_{i},\rho =1,2,\ldots ,m,$$

the CME that describes this CRN should add up all the terms corresponding to each reaction, i.e.,

(10) $$\begin{array}{c} \frac{dP(\vec{n},t)}{dt}=\sum_{\rho =1}^{m}\left\{k_{+}^{\rho }V\left(\prod_{i=1}^{J}E^{s_{i}^{\rho }-r_{i}^{\rho }}-1\right)\prod_{j=1}^{J}\left[\frac{n_{j}!}{\left(n_{j}-s_{j}^{\rho }\right)!V^{s_{j}^{\rho }}}\right]\right.\;\;\;\;\;\;\;\;\;\;\;\\ \left.+k_{-}^{\rho }V\left(\prod_{i=1}^{J}E^{r_{i}^{\rho }-s_{i}^{\rho }}-1\right)\prod_{j=1}^{J}\left[\frac{n_{j}!}{\left(n_{j}-r_{j}^{\rho }\right)!V^{r_{j}^{\rho }}}\right]\right\}P. \end{array}$$

**Figure 1:**
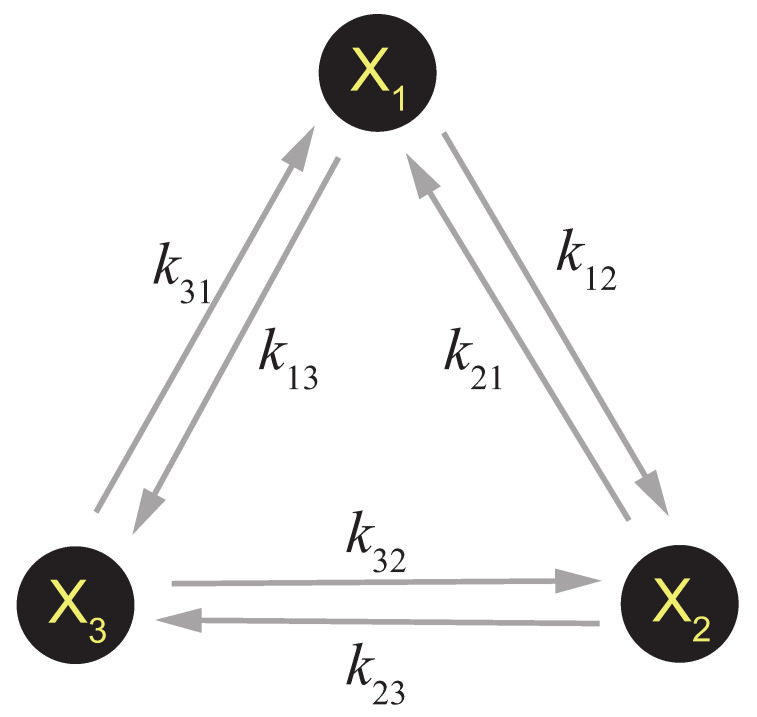
A simple 3-component unimolecular CRN.

This is how to describe a general CRN. Sometimes this can be simplified. If the reaction system only consists of a series of unimolecular reactions (for example, a CRN that describes the decoration or allosteric transition of a single protein complex), a simplified framework can be applied [36]. As is shown in Figure 1, suppose there is a system consisting of three components $X_{1},X_{2},X_{3}$ which can be transferred to each other by unimolecular reactions, i.e.,

$$X_{1}\overset{k_{12}}{\underset{k_{21}}{⇌}}X_{2}\overset{k_{23}}{\underset{k_{32}}{⇌}}X_{3}\overset{k_{31}}{\underset{k_{13}}{⇌}}X_{1}.$$

The dynamics of this system can be regarded as a single molecule jumping between the three states $X_{1},X_{2},X_{3}$, with the probability stopping at each state $p_{i}$ equals to the concentration of each component at steady state. Thus the evolution of $p_{i}$ is governed by the following master equation:

(11) $$\frac{dp_{i}}{dt}=\sum_{j\neq i}\left(k_{ji}p_{j}-k_{ij}p_{i}\right),$$

which has a much simpler form than the general Equation (10).

In summary, generally the dynamics of a CRN can be regarded the system jump between different microscopic states via Markov processes and can be described by a CME. Actually, such description in terms of stochastic process is proposed much earlier than the research on NESS or stochastic thermodynamics and had already brought in many important applications. For example, the precise simulation of chemical reactions—Gillespie algorithm [43].

### 3.2. Thermodynamic Quantities in Chemical Reaction Networks Out of Equilibrium

How to define the thermodynamic quantities in chemical reaction systems? Although they are well defined in equilibrium statistical mechanics, these definitions are traditionally only proved to hold at equilibrium state. Luckily, some of the definitions can be extend to nonequilibrium systems. The extension is strictly established by Ge and Qian [44], which is partly reported below. Their original work ignored the cases where different microstates can be connected by multiple reactions, so the content below is slightly modified. It should be noted that the extension naturally converges to standard equilibrium definitions in thermodynamics, similarly to what happens in other literature [45].

Consider a general CRN, where any two microscopic states *i* and *j* are connected by $\sigma_{ij}$ reactions. The dynamics of this CRN is governed by the following CME:

(12) $$\frac{dp_{i}}{dt}=\sum_{j}\sum_{\alpha =1}^{\sigma_{ij}}\left(q_{ji}^{\alpha }p_{j}-q_{ij}^{\alpha }p_{i}\right),i=1,2,\ldots$$

with $p_{i}$ the probability at state *i* and $q_{ji}^{\alpha }$ the reaction rate resulted from α-th reaction that switch the system from state *i* to *j*. Mathematically, it can be proved that for a quite wide range of CRN, there exists a unique steady state distribution $p_{i}^{s}$. Hence the internal energy for microstates can be defined with self-consistency:

(13) $$u_{i}\equiv -k_{B}T\ln p_{i}^{s},$$

and total internal energy is $U=\sum_{i}p_{i}u_{i}$. The entropy can be defined by Gibbs entropy:

(14) $$S\equiv -k_{B}\sum_{i}p_{i}\ln p_{i},$$

thus the free energy is

(15) $$F\equiv U-TS=-k_{B}T\sum_{i}p_{i}\ln\frac{p_{i}}{p_{i}^{s}}.$$

By taking time derivatives to *F* and *S*, it can be determined how free energy and entropy vary over time:

(16) $$\frac{dF}{dt}=-\frac{1}{2}k_{B}T\sum_{i,j}\sum_{\alpha =1}^{\sigma_{ij}}\left(p_{i}q_{ij}^{\alpha }-p_{j}q_{ji}^{\alpha }\right)\ln\left[\frac{p_{j}\left(t\right)p_{i}^{s}}{p_{i}\left(t\right)p_{j}^{s}}\right],$$

and

(17) $$\frac{dS}{dt}=e_{p}-\frac{h_{d}\left(t\right)}{T},$$

where

(18) $$\begin{array}{c} e_{p}=\frac{1}{2}k_{B}\sum_{i,j}\sum_{\alpha =1}^{\sigma_{ij}}\left(p_{i}q_{ij}^{\alpha }-p_{j}q_{ji}^{\alpha }\right)\ln\frac{p_{i}q_{ij}^{\alpha }}{p_{j}q_{ji}^{\alpha }}, \end{array}$$

(19) $$\begin{array}{c} h_{d}=\frac{k_{B}T}{2}\sum_{i,j}\sum_{\alpha =1}^{\sigma_{ij}}\left(p_{i}q_{ij}^{\alpha }-p_{j}q_{ji}^{\alpha }\right)\ln\frac{q_{ij}^{\alpha }}{q_{ji}^{\alpha }}. \end{array}$$

$e_{p}$ is the entropy production rate of the chemical reactions, and is never negative. $h_{d}$ is the heat dissipation rate describing how much heat is dissipated and expelled to external environment.

At steady state $p_{i}=p_{i}^{s}$, it’s clear that $dF/dt=dS/dt=0,e_{p}^{s}=h_{d}^{s}/T$. This indicates that, when $e_{p}^{s}=0$, system is at equilibrium and there is no heat exchange with external environment. When $e_{p}^{s}>0$, system is driven to a NESS, continuously dissipating energy with a rate $e_{p}^{s}$ and expelling these entropy to external environment with a rate $h_{d}^{s}/T$ in the form of heat. Therefore, considering energy conservation law, the free energy dissipation rate of the system is

(20) $$\dot{W}=Te_{p}^{s}=\frac{1}{2}k_{B}T\sum_{i,j}\sum_{\alpha =1}^{\sigma_{ij}}\left(p_{i}^{s}q_{ij}^{\alpha }-p_{j}^{s}q_{ji}^{\alpha }\right)\ln\frac{p_{i}^{s}q_{ij}^{\alpha }}{p_{j}^{s}q_{ji}^{\alpha }}.$$

In some literature, it’s also called “housekeeping heat” $Q_{hk}$ [44], with the name suggesting it’s the necessary energy input to maintain such a NESS. In addition, $Q_{hk}>0$ for NESS, which is in accordance with Schrödinger and Prigogine’s theory.

When the system’s microscopic states are so close that can be described by some continuous variables $\vec{x}=\left(x_{1},x_{2},\ldots \right)$ (for example, in thermodynamic limit where the total molecular number goes to infinity, the system is described by its concentration), the CME can be approximated by a Fokker-Planck equation [41,46]:

(21) $$\frac{\partial P(\vec{x},t)}{\partial t}=-\sum_{\alpha }\frac{\partial J_{\alpha }}{\partial x_{\alpha }},$$

with α the reaction index and the sum runs over all the possible chemical reactions. $J_{\alpha }=F_{\alpha }P-D_{\alpha }\partial_{x_{\alpha }}P$ is the chemical flux resulted from α-th reaction, with $x_{\alpha }$ the generalized coordinate corresponding to the direction of this reaction, $F_{\alpha }$ and $D_{\alpha }$ the corresponding probability drift force and diffusion constant. Note that $x_{\alpha }$ may be different from $x_{1},x_{2},\ldots$, because the direction of the reaction may not be parallel to any of $x_{1},x_{2},\ldots$ For example, a system consisting of *n* components can be described by the concentration of each component $c_{i},i=1,2,\ldots ,n$, thus $\vec{x}=\left(c_{1},c_{2},\ldots ,c_{n}\right)$. Suppose there is a reaction $X_{1}⇌X_{2}$, whose forward reaction would equally increase $c_{2}$ and decrease $c_{1}$. The generalized coordinate of this reaction is $c_{2}-c_{1}$, which is not parallel to any of $c_{1},c_{2},\ldots ,c_{n}$.

Similar to Equation (20), it can be proved that the free energy dissipation rate at steady state $P_{s}\left(\vec{x}\right)$ is [47]

(22) $$\dot{W}=k_{B}T\sum_{\alpha }\int \frac{J_{\alpha }^{2}}{D_{\alpha }P_{s}}d\vec{x}.$$

Equation (22) can also be obtained by taking Equation (20) in the limit of state distance going to zero, indicating the two cases are essentially the same.

### 3.3. Cycle Theory and the Break of Detailed Balance

As stated above, generally there would be a steady state in a CRN. However, in different cases these steady states have different physical meanings. If the reaction rates in the CRN satisfies the so-called detailed balance condition, i.e., each forward probability flux from a reaction is nullified by the reverse probability flux from the corresponding reverse reaction, the steady state is the equilibrium state and there is no net probability flux in the system. If the detailed balance condition is not satisfied, net probability flux would arise and entropy would continuously be produced. For the system that doesn’t have a source or a sink, such net flux must be a cyclic flux. There are many work on the such cyclic flux since Hill [48], and it has been widely accepted that the existence of cyclic flux in a system is one of the most important signature for the system to be nonequilibrium.

The system shown in Figure 1 is a simple case to illustrate this concept. By definition, the probability flux of each reaction is $J_{ij}=k_{ij}p_{i}-k_{ji}p_{j},$ and the thermodynamic force is [48]

$$X_{ij}=k_{B}T\ln\frac{k_{ij}}{k_{ji}}+k_{B}T\ln\frac{p_{i}}{p_{j}}=k_{B}T\ln\frac{k_{ij}p_{i}}{k_{ji}p_{j}}.$$

According to Equation (18), the entropy production rate of each chemical reaction is $e_{p,ij}=T^{-1}J_{ij}\times X_{ij}\geq 0$. Denoting the steady state by $p_{i}^{s},i=1,2,3,$ at steady state the fluxes should satisfy $J_{12}=J_{23}=J_{31}\equiv J^{s}$. Hence the total free energy dissipation rate at steady state is

(23) $$\dot{W}=Te_{p}^{s}=k_{B}TJ^{s}\ln\frac{k_{12}k_{23}k_{31}}{k_{13}k_{32}k_{21}}=k_{B}TJ^{s}\ln\gamma \geq 0,$$

with $\gamma \equiv (k_{12}k_{23}k_{31})$/$\left(k_{13}k_{32}k_{21}\right)$.

Each factor in Equation (23) has a quite clear physical meaning. $J^{s}$ is the net cyclic flux in the cycle. When $J^{s}>0$ there will be a clockwise net flux $X_{1}\to X_{2}\to X_{3}\to X_{1}$, and when $J^{s}<0$ there will be a counter-clockwise net flux $X_{1}\to X_{3}\to X_{2}\to X_{1}$. γ is the ratio of the clockwise rate product and the counter-clockwise rate product, and lnγ is the thermodynamic force of the cycle. When detailed balance is satisfied and $k_{ij}p_{i}^{s}=k_{ji}p_{j}^{s}$ holds for every reaction, $J^{s}=0,\ln\gamma =0$, suggesting both the net cyclic flux and the thermodynamic force are 0, thus the entropy production rate is 0 and the system is at equilibrium state. When detailed balance is broken, both the net cyclic flux and the thermodynamic force will arise, γ≠1, with a positive entropy production rate indicating the system is at nonequilibrium state. Hence, γ≠1 is equivalent to the break of detailed balance.

How is the thermodynamic force introduced and detailed balance broken in a realistic systems? Qian illustrated the answer to this question with a simple example in a review [36]. In short, when detailed balance is broken, this network must be coupled with some reactions that involve molecules whose amount is under controlled by external constraints or regulations. For example, in the system shown in Figure 1, suppose the reaction $X_{1}⇌X_{2}$ is actually coupled with an ATP hydrolysis reaction and the real elementary reaction is

$$X_{1}+\mathrm{ATP}\overset{k_{12}^{0}}{\underset{k_{21}^{0}}{⇌}}X_{2}+\mathrm{ADP},$$

where inorganic phosphate Pi can be considered as a constant and is ignored. The reaction rates and the original rates are related by $k_{12}=k_{12}^{0}\left[ATP\right]$, $k_{21}^{0}=k_{21}\left[ADP\right]$. By definition, the reaction potential of each reaction is $\Delta \mu_{ji}=-k_{B}T\ln\left(k_{ij}/k_{ji}\right),$ and it’s easy to show that the thermodynamic force over a cycle $\Delta \mu_{cycle}\equiv \Delta \mu_{12}+\Delta \mu_{23}+\Delta \mu_{31}=k_{B}T\ln\gamma$. In absence of external regulation, the system is closed and naturally should satisfy detailed balance, thus $\Delta \mu_{cycle}=0$ and

(24) $$\gamma^{eq}=\frac{k_{12}^{0}k_{23}k_{31}{\left[ATP\right]}^{eq}}{k_{13}k_{32}k_{21}^{0}{\left[ADP\right]}^{eq}}=\frac{k_{12}^{eq}k_{23}k_{31}}{k_{13}k_{32}k_{21}^{eq}}=\exp\left(\frac{\Delta \mu_{cycle}}{k_{B}T}\right)=1.$$

when the external regulation takes place and fixes the concentration ratio [ATP]/[ADP] to a constant value different from ${\left[ATP\right]}^{eq}/{\left[ADP\right]}^{eq}$, the rate ratio $k_{12}/k_{21}$ will be effectively tuned, thus

(25) $$\gamma =\frac{k_{12}k_{23}k_{31}}{k_{13}k_{32}k_{21}}=\frac{k_{12}^{0}k_{23}k_{31}{\left[ATP\right]}^{eq}}{k_{13}k_{32}k_{21}^{0}{\left[ADP\right]}^{eq}}\cdot \frac{\left[ATP\right]{\left[ADP\right]}^{eq}}{\left[ADP\right]{\left[ATP\right]}^{eq}}=\frac{\left[ATP\right]{\left[ADP\right]}^{eq}}{\left[ADP\right]{\left[ATP\right]}^{eq}}\neq 1,$$

and $\Delta \mu_{cycle}\neq 0.$ Therefore, for a general reaction coupled with ATP hydrolysis, e.g., phosphorylation, by tuning the concentration ratio between ATP and ADP, the thermodynamic force on a cycle $\Delta \mu_{cycle}$ could be tuned and detailed balance can be broken. In addition, it can be checked that

(26) $$\ln\gamma =\ln\frac{\left[ATP\right]{\left[ADP\right]}^{eq}}{\left[ADP\right]{\left[ATP\right]}^{eq}}=\frac{\Delta \mu_{ATP,0}}{k_{B}T}+\ln\frac{\left[ATP\right]}{\left[ADP\right]}=\frac{\Delta \mu_{ATP}}{k_{B}T},$$

where $\Delta \mu_{ATP,0}$ is the ATP hydrolysis energy under standard condition. Therefore, $\Delta \mu_{cycle}$ is exactly the free energy that one ATP molecule can produce by hydrolysis at the condition. This result explicitly shows how external energy source is pumped in to drive the system, and is interesting and quite important.

The above discussion can be extended to arbitrary CRNs without sink or source. If there is no cycle in the network, detailed balance will never be broken and the steady state is always equilibrium state. If there are more than one cycle, these cycles can be decomposed to a series of linear independent basis cycles $C_{i}$, whose ratio of the rate products in clockwise reactions and counter-clockwise reactions $\gamma_{i}$ determines whether detailed balance is broken on the basis cycle and the corresponding thermodynamic force. The net flux in the system at steady state can also be decomposed into a series of cyclic fluxes $J_{c_{i}}^{s}$, and the total free energy dissipation rate is adding up all the dissipation on each basis cycle, i.e., $k_{B}T\sum_{i}J_{c_{i}}^{s}\ln\gamma_{i}$. More details about the cycle decomposition can be found in a review by Schnakenberg and some proceeding literature [49,50,51,52]. From their work, it turns out that such decomposition of thermodynamic observables into cycles might be even more fundamental than transition themselves.

At the end of this section, we note that it’s also possible to study the CRN by deterministic thermodynamics and don’t need to concern stochastic theory. For example, the above cycle theory can be obtained merely by deterministic methods, as is shown in the work done by Rao and Esposito [52]. In the framework of mean-field thermodynamics, by analyzing the stoichiometric matrix of the CRN with method from linear algebra, the conservation law and basis cycles can be calculated. And by calculating the chemical potential difference $\Delta \mu =k_{B}T\ln\gamma$ on each basis cycle, the thermodynamic force can be determined. The dynamics of the system is governed by law of mass action, so the steady state flux can also be determined. Hence, the total dissipation rate can be calculated just like above, which is in accordance with stochastic theory. In addition, such methodologies from mean-field thermodynamics are also capable to determine whether the detailed balance is broken in a CRN with irreversible reactions, for example the work done by Gorban and collaborators [53,54]. Their results pointed out that irreversible reactions cannot be involved in any cycles to prevent divergence of entropy production, and are also in accordance with stochastic theory. Some other notable results are obtained in the last decades following similar methodology [55,56,57,58,59,60,61,62]. This mean-field methodology to study thermodynamics is traditionally Brussels School would use.

The advantage to adopt the stochastic theory is that we can calculate not only the thermodynamic quantities themselves such as entropy production rate but also their uncertainties and errors. This is critical in living systems, since the system size is usually small and fluctuation or noise may play an important role in various biological mechanisms [63,64,65]. And such fluctuation is deeply related to entropy production rate with TUR Equations (5) and (6) mentioned above. It shows that, no matter how to design the parameters in a CRN, it’s impossible to suppress the relative fluctuation and entropy production rate to infinitely small at the same time.

## 4. Thermodynamics for Information Processing in Living Systems

In realistic systems, the form of maintaining the steady state by dissipation free energy is to fulfill various biological functions by burning molecular fuels. Some functions directly transfer the chemical energy in the fuel to mechanical energy, for example the famous molecular motor [66,67,68,69,70,71] which plays an important role in translocation in cells and motion of bacteria. Some functions involve the synthesis of complex molecules [72,73,74,75,76], e.g., DNA and protien complex.

Except these functions where the energy have clear physical or chemical purposes, there are another large group of functions that only involve in the signal transduction processes. These functions are called information processing functions. The signal is usually transduced by a series of pathways and networks, where the allosteric transition and modification (methylation, phosphorylation, ubiquitination, etc.) to the signal molecule play the important role. For example, the key step in the chemotaxis network in *E.coli* is the methylation and demethylation of the chemoreceptor dimer [77]. Such modification processes are usually accompanied by hydrolysis of the energy molecules (ATP, GTP, etc.), hence free energy will be dissipated when the information is processed.

A natural question is, from the physics point of view, why free energy dissipation is needed during information processing? The hint comes from the development of information theory in modern statistical physics, especially a branch called “information thermodynamics” [55,78,79,80,81] which studies the thermodynamic cost to manipulate information or vise versa. Hence, an intuitive understanding is that the free energy dissipation is the necessary cost to process the information, or could be used to improve the processing accuracy. Actually, there has been some pioneer works revealing the cost for different information processing functions, suggesting this understanding should generally be right. Here we show some typical results for examples.

### 4.1. The Accuracy of Specificity and Kinetic Proofreading

The study on the accuracy of specificity and kinetic proofreading is one of the earliest study that applies nonequilibrium thermodynamic concepts to information processing in living systems. This study could trace back to Hopfield’s work in 1974 [82], which has already become a classical research paradigm in this area.

This problem arises from the synthesis of complex molecules, such as DNA replication. As is widely known, successful DNA replication is achieved through base paring. For a certain site in the template DNA strand, the affinity of the “correct” base that should be paired to this site is much larger than the “wrong” base. This property that the specific ligand could bind to specific substrate or receptor is the so-called specificity. However, although small, the probability of a wrong base pair is not exactly zero. By calculating the free energy difference between correct and wrong base pairs, researchers found that, if the affinity difference is the only reason leading to specificity, the error rate during DNA replication is around $10^{-4}$∼$10^{-5}$. This error rate is too large for gene to maintain its relative invariance. But actually, in realistic cases, for eukaryotes the error rate is only around $10^{-9}$ [82], much smaller than the estimation by affinity. Hence there must be some other mechanism to reduce the error, which is interesting to researchers for long.

Under this circumstance, Hopfield proposed a so-called kinetic proofreading model [82]. This model supposes that there are one receptor and two ligands in the model, one “correct” and one “wrong”. The affinity difference between the two ligands at equilibrium leads to a basic binding error rate $f_{0}$. On top of that, if an irreversible “proofreading reaction” is introduced, dissociating the ligand from the receptor and allowing the receptor to choose the ligand again, the error rate could be further reduced. In this case, the system is driven out of equilibrium because the “proofreading reaction” breaks detailed balance. The error rate could be even reduced to $f_{0}^{2}$ under some specific conditions, which well explains the small error rate during DNA replication.

**Figure 2:**
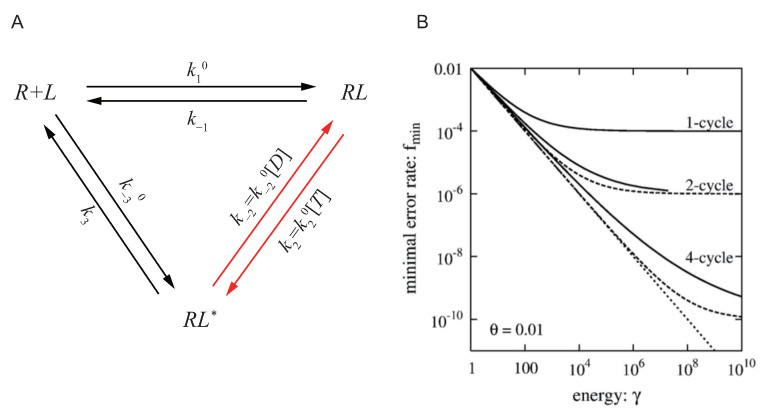
Thermodynamic limitation of specificity and kinetic proofreading, adopted from Ref. [77]. (**A**) The cycle for the reactions between receptor *R* and ligand *L*. *R* could bind with *L* forming the complex RL, and RL could be activated to $RL^{*}$. The activated $RL^{*}$ could dissociate to free ligand *L* and receptor *R*. The activation of RL is usually achieved by modification such as phosphorylation, and phosphorylation is usually coupled with ATP hydrolysis, so this reaction arrow is colored red indicating it’s where the external energy is put in. *T* stands for ATP and *D* stands for ADP in the panel, with Pi not presented. (**B**) The accuracy of specificity is limited by energy from ATP hydrolysis. The solid lines show the relation between the minimal error rate $f_{min}$ with different number of proofreading cycles and the energy parameter γ. It shows that, when the energy of ATP hydrolysis $\Delta \mu_{ATP}=k_{B}T\ln\gamma$ is fixed, no matter how to design the structure of the proofreading reaction networks, the thermodynamic limitation Equation (33) illustrated by dotted lines cannot be broken.

In Hopfield’s work, due to the limitation of the development of thermodynamic theory at that time, he didn’t carefully discuss the relation between the error rate reduction and how far the system is driven out of equilibrium. In a more recent work in 2006, Qian discussed the same question with more details [83]. As is shown in Figure 2A, Hong presented Hopfield’s model in a more rigorous way with nonequilibrium thermodynamics and cycle theory. Ligand *L* can bind with receptor *R* forming the complex RL, and RL could be activated to $RL^{*}$ by hydrolyzing ATP. The activated $RL^{*}$ could then dissociate to free ligand *L* and receptor *R*. Denoting the ligand concentration by [L], the rate product ratio is

(27) $$\gamma =\frac{k_{1}^{0}\left[L\right]k_{2}k_{3}}{k_{-1}k_{-2}k_{-3}^{0}\left[L\right]}=\frac{k_{1}k_{2}k_{3}}{k_{-1}k_{-2}k_{-3}},$$

with $k_{1}=k_{1}^{0}\left[L\right],k_{-3}=k_{-3}^{0}\left[L\right]$. As discussed in previous section, it can be proved that $k_{B}T\ln\gamma$ is exactly the energy to hydrolyze one ATP molecule. When the concentration ration of ATP and ADP [T]/[D] is large enough, γ>1, ATP can be effectively hydrolyzed, and the system is driven out of equilibrium.

On top of that, suppose there are two ligands $L^{\prime }$ (the correct ligand) and *L* (the wrong ligand) in the system with the same concentration. Their structures are similar (so have the same $k_{1},k_{2},k_{-2},k_{-3}$) but $L^{\prime }$ has a much larger affinity than *L*. This leads to a smaller dissociation rate than *L*:

$$\frac{k_{-1}^{\prime }}{k_{-1}}=\frac{k_{3}^{\prime }}{k_{3}}\equiv \theta <1.$$

The error rate can be defined by the affinity ratio for the two ligands at activated state,

(28) $$f=\frac{\left[RL^{*}\right]/\left(\left[R\right]\left[L\right]\right)}{\left[R{L^{\prime }}^{*}\right]/\left(\left[R\right]\left[L^{\prime }\right]\right)},$$

It can be proved that, at equilibrium state f=θ. When the system is driven out of equilibrium, γ>1, *f* can be smaller than θ. It can be calculated that, for a fixed γ, the minimal error rate by tuning other parameters is

(29) $$f_{min}\left(\gamma \right)=\theta {\left(\frac{1+\sqrt{\gamma \theta }}{\sqrt{\gamma }+\sqrt{\theta }}\right)}^{2}.$$

It can be checked that $f_{min}\left(\gamma \right)$ monotonically decreases with γ, and when γ→∞, $f_{min}\to \theta^{2}$, which is exactly Hopfield’s result.

In this paper, Hong also proposed that γ gives a universal thermodynamic limitation to the error rate $f_{min}$, which would not be violated no matter what structure the reaction network has. The essence of the specificity is the competitive binding between the two ligands and one receptor, which can be represented by a single reaction:

(30) $$L+R{L^{\prime }}^{*}⇌L^{\prime }+RL^{*},$$

with the equilibrium constant equals to θ. The error rate can be defined as

(31) $$f=\frac{\left[RL^{*}\right]}{\left[R{L^{\prime }}^{*}\right]}.$$

Given that the concentration of *L* and $L^{\prime }$ are equal, the free energy difference between $RL^{*}$ and $R{L^{\prime }}^{*}$ at steady state is

(32) $$\Delta \mu =-k_{B}T\ln\theta +k_{B}T\ln\frac{\left[RL^{*}\right]}{\left[R{L^{\prime }}^{*}\right]}=k_{B}T\ln\frac{f}{\theta }.$$

when at equilibrium, Δμ should be zero and f=θ. If this reaction is coupled to a ATP hydrolysis reaction and driven out of equilibrium so that $\Delta \mu =-\Delta \mu_{ATP}=-k_{B}T\ln\gamma ,$γ≠1, we have the following relation

(33) $$f=\frac{\left[RL^{*}\right]}{\left[R{L^{\prime }}^{*}\right]}=\frac{\theta }{\gamma }.$$

This is the thermodynamic limit given by γ.

As is shown in Figure 2A, different solid lines illustrate the relation between the minimal error rate $f_{min}$ with different number of proofreading cycles and the energy parameter γ. The solid lines all lie above the dotted line representing the thermodynamic limit Equation (33), confirming that the accuracy of specificity is limited by the thermodynamic cost.

### 4.2. The Accuracy of Oscillators and the Energy Cost

Oscillatory behaviors exist in many biological systems and are crucial in controlling the timing of various living activities. However, due to noise, any oscillator cannot keep its high accuracy forever. The time that the oscillator takes to complete one cycle is never exactly equal to the mean period. Affected by noise, it would be slightly longer or shorter. With more cycle completed, the error will accumulate more and more, and finally lost the function of timing. In terms of physics, after a long time, the phase of the oscillator will lost its coherence to initial phase. How to overcome the noise and maintain coherence in a relatively long time, is an important question.

**Figure 3:**
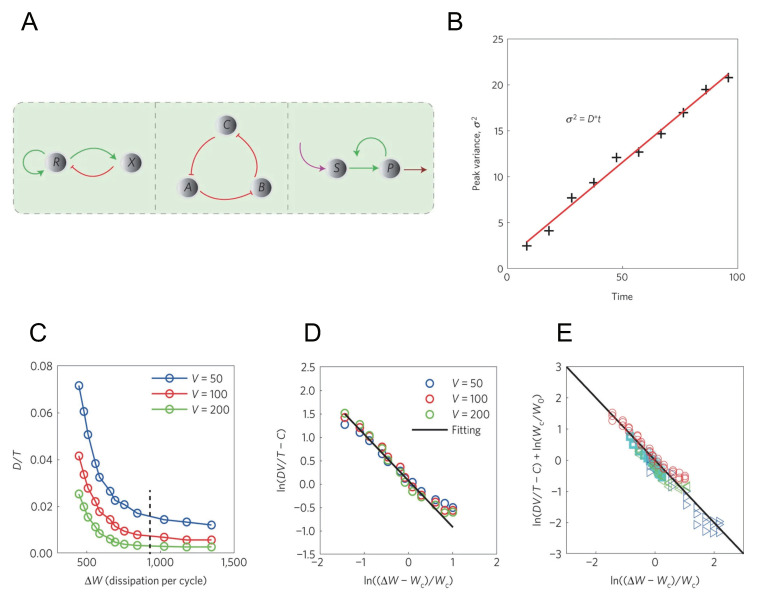
The relation between the coherence in the accurate oscillation network and its energy cost, adopted from Ref. [84]. (**A**) Three motifs that could generate oscillation. Left panel: activator-inhibitor model. Middle panel: repressilator model. Right panel: substrate-depletion model. In Ref. [84], both Brusselator and glycolysis are classified to substrate-depletion model. (**B**) Definition of phase diffusion constant *D*. The black crosses label the variance of the peak time which grows linearly, and the slope is *D*. (**C**) The relation between *D* in activator-inhibitor model and the energy dissipation in a period ΔW. The black dashed line corresponds to the energy of one ATP molecule hydrolysis $\Delta \mu_{ATP}\approx 12k_{B}T$. (**D**) is (**C**) in log scale after normalized by volume *V*. The black solid line is the fitting result from Equation (34). (**E**) The fitting result for all the for models after normalized by volume *V*. All the simulation results collapse to the same fitting line (black solid line). Different marks represent results from different models: red circle for activator-inhibitor model, cyan square for repressilator, blue triangle for Brusselator, green triangle for glycolysis.

In Ref. [84], researchers thoroughly studied this problem. As is shown in Figure 3A, researchers enumerated all the possible motifs that could generate oscillation in three-node networks, including activator-inhibitor model, repressilator and substrate-depletion model. Researchers provided three realistic examples for each of the three motifs, along with a famous Brusselator model (which actually belongs to substrate-depletion model) and studied the oscillation accuracy and the energy cost for the respective cases.

As is shown in Figure 3B, researchers defined a phase diffusion constant *D* to describe the coherence of the oscillatory network. The results reveal that all the four models can oscillate only when far from equilibrium, and the dimensionless phase diffusion constant D/T (*T* is the oscillation period) inversely depends on the energy dissipation in a period ΔW:

(34) $$V\times \frac{D}{T}\approx C+\frac{W_{0}}{\Delta W-W_{c}},$$

with $W_{c}$ a critical energy, *V* the system volume, *C* and $W_{0}$ two constants. This equation shows that the accuracy of the oscillators inversely depends on the additional energy to the minimal requirement for oscillation, and is used to fit the simulation of the 4 models, as is shown in Figure 3C–E. From the fitting, it turns out that Equation (34) is applicable for all the four models.

To understand the origin of this inverse dependence, researchers consider the noisy Stuart-Landau equation that describes a general system near Hopf bifurcation:

(35) $$\frac{dr}{dt}=ar-cr^{3}+\eta_{r},\frac{d\theta }{dt}=b+dr^{2}+\eta_{\theta },$$

where $\eta_{r},\eta_{\theta }$ are noise terms satisfying

$$\langle \eta_{r}\left(t\right)\eta_{r}\left(t^{\prime }\right)\rangle =2\Delta \delta \left(t-t^{\prime }\right),\langle \eta_{\theta }\left(t\right)\eta_{\theta }\left(t^{\prime }\right)\rangle =\frac{2\Delta \delta }{r^{2}}\left(t-t^{\prime }\right).$$

In this system, by calculating the energy dissipation and phase diffusion constant with parameters a,b,c,d,Δ in Equation (35), Equation (34) can be analytically obtained when a≪bc/d, and $C,W_{0},W_{c}$ can be expressed by:

$$C=0,W_{c}=\frac{8\pi d}{c}>0,W_{0}=\left(\frac{d^{2}}{2c}+2c\right)\left(\frac{2\pi b}{c\Delta }+\frac{2\pi d^{2}}{bc^{2}}\right)\Delta ,$$

hence the inverse relation Equation (34) holds in a quite large range and is relatively general.

In some biological systems, timing function is accomplished by the collective oscillation phase of a group of oscillating molecules. For such systems, synchronization among different molecular oscillators is of great importance to generate and maintain the correlation of the collective phase, which is critical for macroscopic oscillation accuracy. A recent work shows that the maximal achievable synchrony among a group of identical molecular oscillators is also inversely dependent on the additional energy cost [85].This result, along with Equation (34), shows that oscillation accuracy can be improved by additional energy both in single oscillator level and collective level.

## 5. Outlook

In this review, we summarize the history and current research status of biological thermodynamics. It’s still a new research branch and hotspot to study the thermodynamics in living systems, which could lead to great fundamental improvement in both physics and biology.

In terms of physics, living systems are realistic systems far from equilibrium, which is a good research object for nonequilibrium statistical physics. By studying the biological functions and their thermodynamic cost, as the examples we show above, the availability of the theories from nonequilibrium statistical physics can be examined by realistic cases. Furthermore, with such applications in realistic systems, physicists could accumulate intuitive feelings that which concepts are of more importance, so that they are able to develop more realistic and useful thermodynamic theories and instruments. It’s also possible to adopt experimental tools from biological systems to test physical theoretical results, just like the test for Jarzynski equality [28,29,30] and fluctuation theorems [31,32,33].

In terms of biology, theoretical tools from thermodynamics and statistical physics could provide another new angle to understand the general, fundamental laws in living systems. New paradigms can also be established with the new theoretical tools to study quantitative behavior in living systems, such as spatial positioning by self-organization [86], understanding fluctuation in cell growth [87] and characterizing enzymes as active matter [88]. In addition, laws from thermodynamics could also help answer the design principles of biological systems, which might be useful to synthetic biology [89,90,91,92] and other biological engineering with in silico design.
